# Hemorrhagic Bullous Lichen Sclerosus of the Breast: A Rare Complication of Radiotherapy

**DOI:** 10.7759/cureus.70194

**Published:** 2024-09-25

**Authors:** Landon Hobbs, Nitin Tiwari

**Affiliations:** 1 Dermatology, University of Virginia School of Medicine, Charlottesville, USA; 2 Dermatology, Adena Medical Center, Chillicothe, USA

**Keywords:** bullous lichen sclerosus, cutaneous adverse effects, medical dermatology, radiotherapy (rt), treatment sequalae

## Abstract

Cutaneous side effects from radiotherapy are commonly reported in breast cancer patients. Radiation-induced hemorrhagic bullous lichen sclerosus (RHBLS) of the breast is a rare, but important, complication of radiotherapy. RHBLS typically presents as painful, hemorrhagic bullae with surrounding sclerotic tissue. A 71-year-old female with a history of whole breast radiotherapy for invasive ductal carcinoma of the right breast and ductal carcinoma in situ (DCIS) of the left breast presented to the clinic with pruritic, firm, and erythematous plaques involving the inframammary folds that progressed into large, painful, hemorrhagic bullae surrounded by porcelain-white skin over several weeks. Biopsy was consistent with RHBLS. She was initially treated with systemic steroids and Clobetasol 0.05% ointment twice daily with partial improvement. To the best of our knowledge, only three definitive cases have been previously reported in the literature making this the fourth case.

## Introduction

Lichen sclerosus (LS) is a chronic inflammatory skin disease characterized by atrophic, white-to-porcelain papules and plaques that can cause significant pruritus and discomfort. Although genetic and autoimmune components have been suspected in the pathophysiology of LS, trauma-induced LS has also been reported [1,2]. The first report of LS affecting the breasts following radiotherapy was published in 1985 [3]. The first case of radiation-induced hemorrhagic bullous LS (RHBLS), a rare subtype of radiation-induced LS characterized by flaccid bullae and purpuric hemorrhage, involving the breasts was reported in 1994 [4]. Herein and to the best of our knowledge, we report the fourth known case of RHBLS of the breasts in a patient with a history of radiotherapy-treated breast cancer and emphasize the importance of early recognition, evaluation for genital involvement, and treatment to avoid further complications. As only three other cases have been documented, only limited conclusions can be drawn, emphasizing the importance of continued investigation into RHBLS.

This article was previously presented as a meeting abstract at the American Academy of Dermatology's 2022 Gross and Microscopic Symposium.

## Case presentation

**Figure 1:**
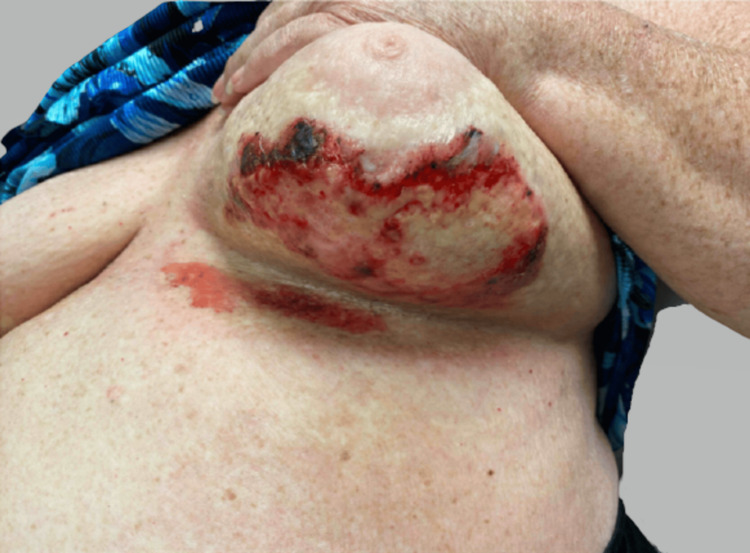
Clinical Photo Hemorrhagic bullae with surrounding sclerotic tissue on the underside of the left breast.

**Figure 2:**
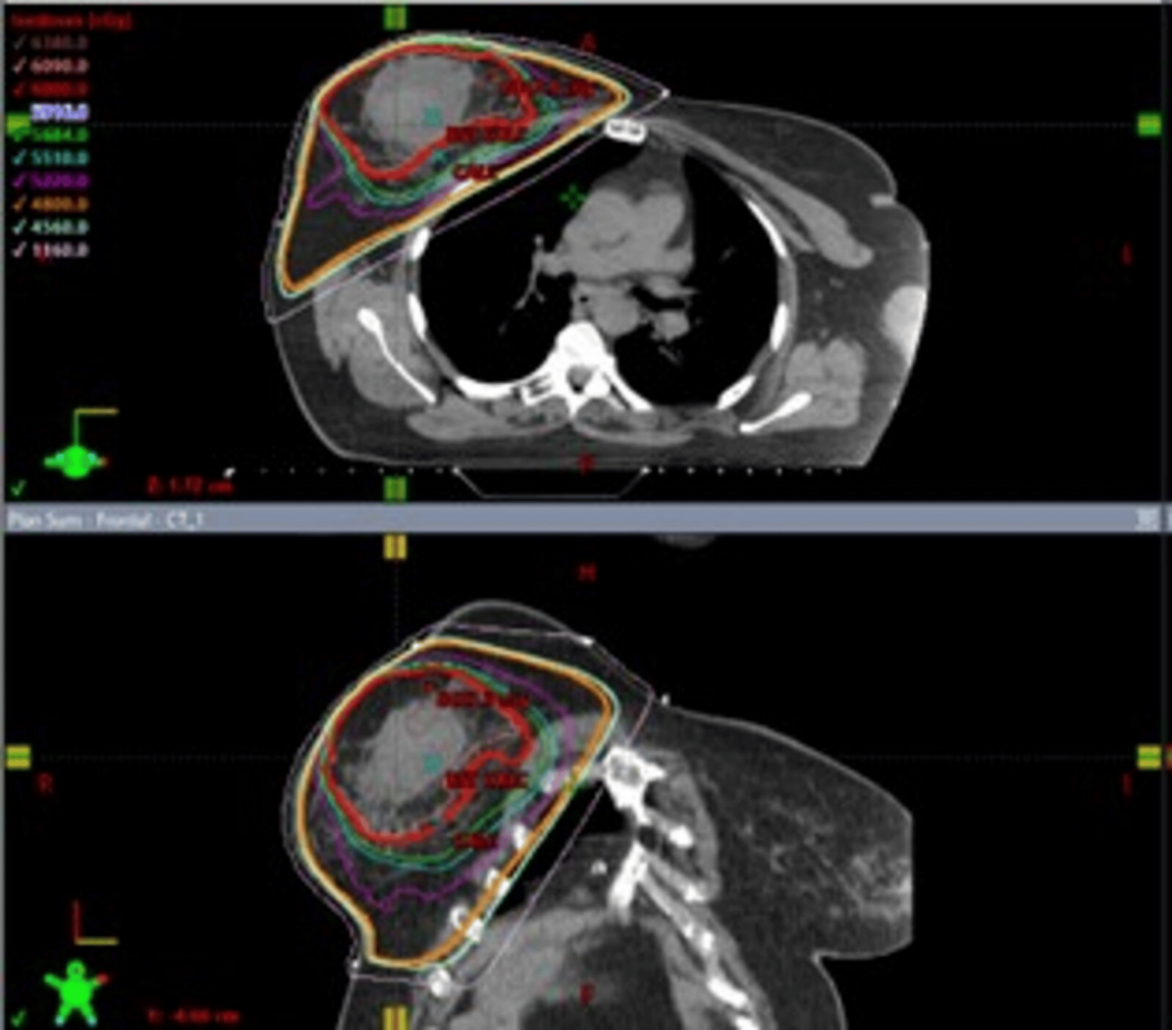
Right Breast Radiotherapy Field Right whole breast radiotherapy in 2011; dosage: 4,800 cGy with 1,200 cGy boost.

**Figure 3:**
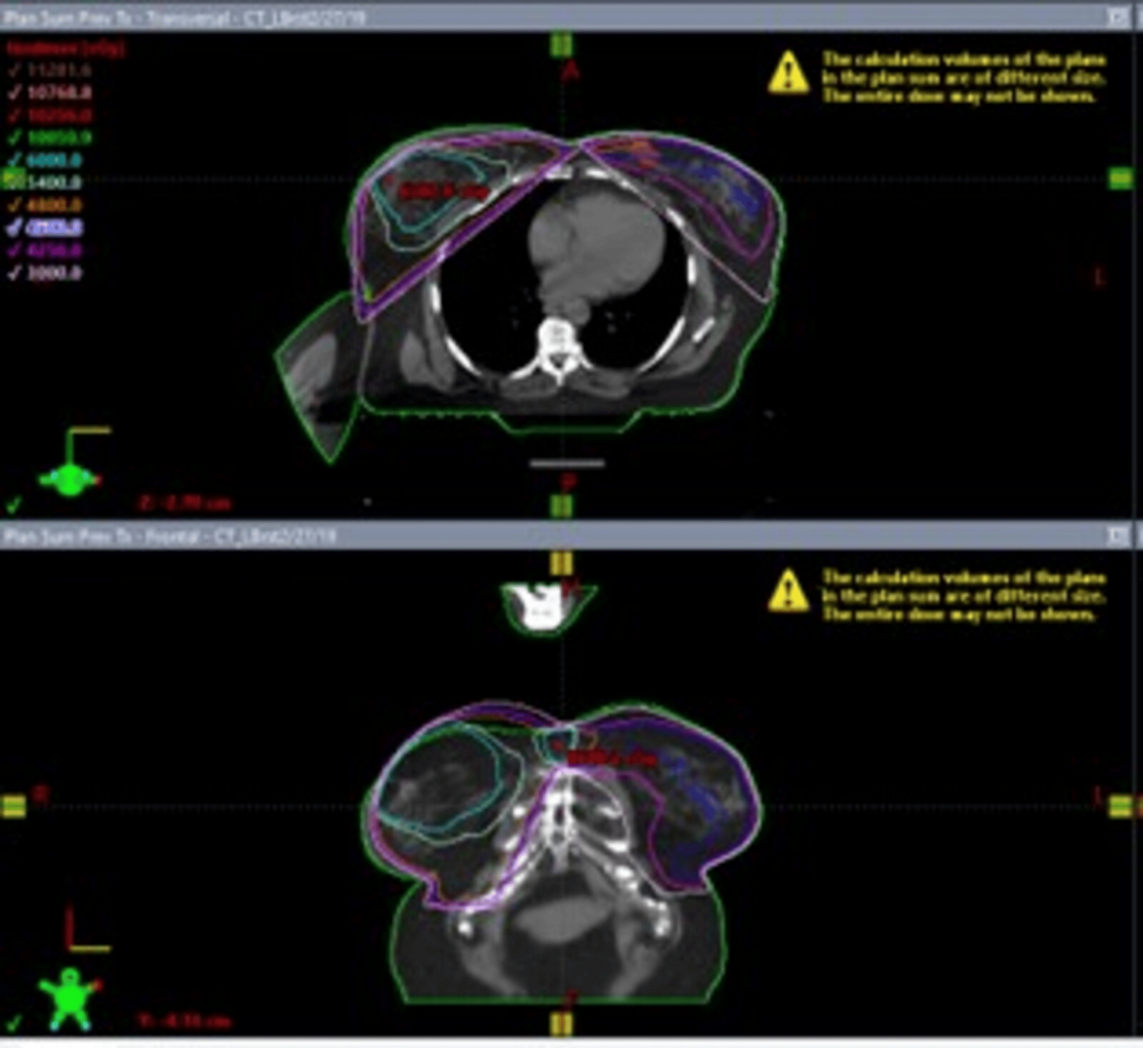
Left Breast Radiotherapy Field Left whole breast radiotherapy in 2019; dosage: 4,256 cGy with 1,000 cGy boost.

**Figure 4:**
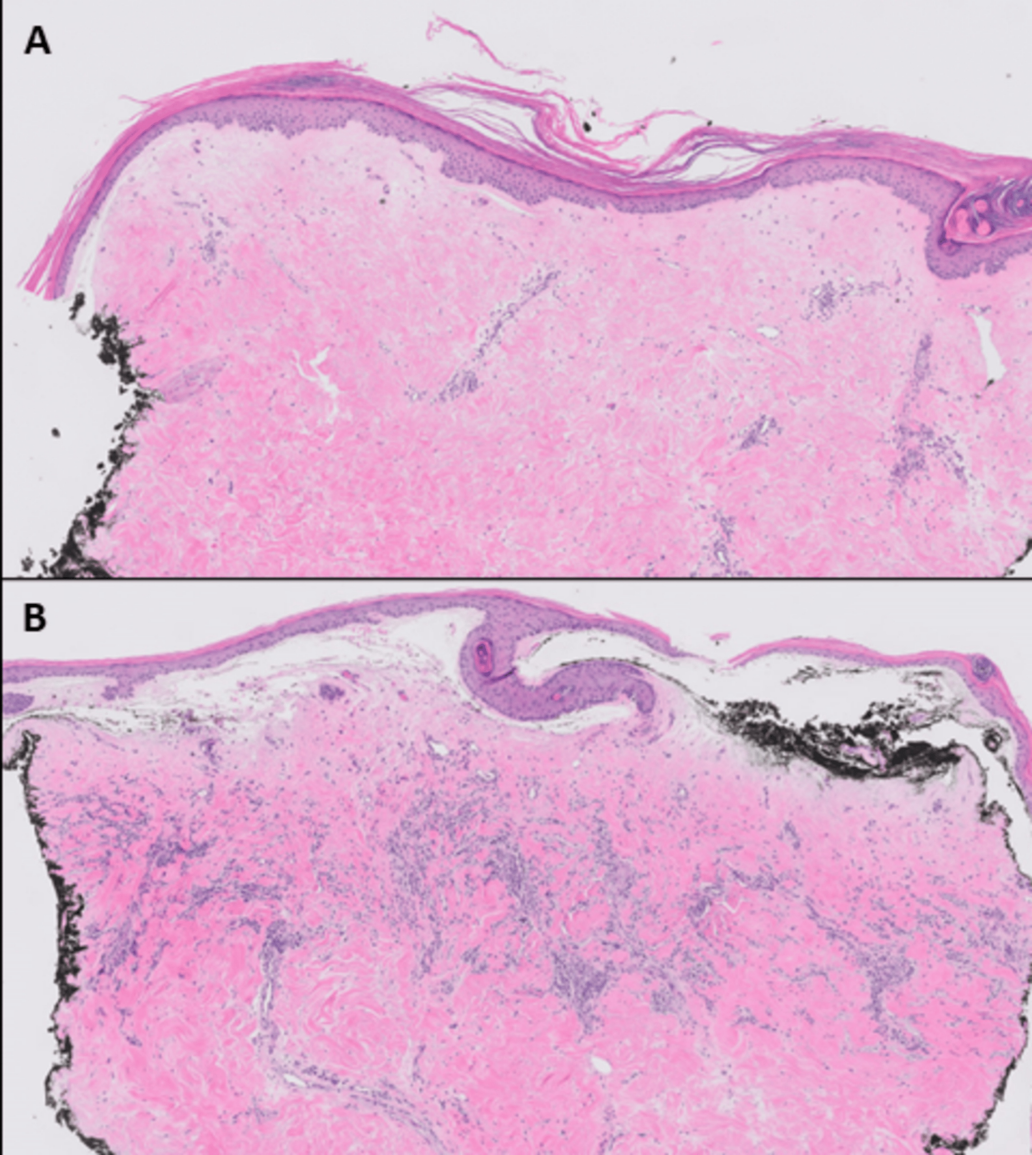
Histopathologic Photos Histopathology of bullous lichen sclerosus (hematoxylin and eosin, 40x). The punch biopsies of sclerotic skin from the left breast (A) and a bulla from the upper abdomen (B) show epidermal atrophy with orthokeratosis, a subepidermal bulla, and a band of edematous, homogenized collagen in the papillary dermis with a dense degree of lymphocytic infiltration.

A 71-year-old female with a history of invasive ductal carcinoma of the right breast (2011) and ductal carcinoma in situ (DCIS) with microinvasive carcinoma of the left breast (2019), cholelithiasis, hyperlipidemia, and carpal tunnel syndrome presented in March 2021 with pruritic, firm, and erythematous plaques in both inframammary folds. Treatment for suspected intertrigo with a combination of topical steroids, nystatin, and zinc oxide was unsuccessful. Over the ensuing weeks, the breasts, as well as the inframammary folds, developed progressively large, painful, hemorrhagic bullae surrounded by porcelain-white skin as noted on physical exam (Figure 1). She was noted to have sclerotic changes in regions outside the field of radiation, including the axillae and inguinal folds. For the invasive ductal carcinoma of the right breast, the patient underwent a partial mastectomy, followed by whole breast radiotherapy (4,800 grays (Gy) with 1,200 centiGrays (cGy) boost) in 2011 (Figure 2) and was treated with anastrozole from 2011 to 2016. In 2019 after an abnormal mammogram, she was found to have DCIS with microinvasive carcinoma of the left breast that was treated with lumpectomy, followed by whole breast radiotherapy (4,526 Gy with 1,000 cGy boost) of the left breast, as well as some lower radiation to the right breast (Figure 3), and letrozole therapy. Given the history of radiotherapy and physical exam findings, biopsies were performed to evaluate for RHBLS, bullous morphea, angiosarcoma, and chronic radiation dermatitis. The biopsies of a hemorrhagic bulla and sclerotic skin from the left breast resulted in a pauci-immune subepidermal bulla and lichen sclerosus, respectively (Figure 4). Taken collectively with the clinical exam, the patient was diagnosed with RHBLS. She was initially treated with systemic steroids and clobetasol 0.05% ointment bis in die (BID) with partial improvement at follow-up two months later.

## Discussion

Cutaneous side effects from radiotherapy have been reported in up to 90% of breast cancer patients [5]. RHBLS of the breast is a rare, yet significant, complication of radiotherapy. To our knowledge, only three definitive cases have been previously reported in the literature [2,4,5] (Table 1). Trattner et al. reported a case of RHBLS of the breast of a 57-year-old female diagnosed with infiltrating ductal carcinoma three years after radiotherapy at a site of previously diagnosed morphea [4]. Peterson et al. reported a case of a 57-year-old female with unilateral RHBLS that presented three months after radiotherapy for invasive ductal carcinoma and eventually progressed to involve both breasts and vulva despite no radiotherapy to the left breast or vulva [5]. Bonfill-Ortí et al. reported RHBLS involving the right breast of a 71-year-old female two years after radiotherapy and letrozole for infiltrating the lobular carcinoma of the right breast [2]. Walsh et al. reported a case of hemorrhagic bullous LS in a 65-year-old woman with a history of breast cancer; however, there was no mention of any history of radiotherapy [6]. As radiotherapy is commonly used as primary and adjuvant treatment in cancer, it is likely that their case also represents RHBLS.

**Table 1:** Cases of Radiation-Induced Bullous Lichen Sclerosus on the Breast Reported in the Literature F, female; OCS, oral corticosteroids; TCS, topical corticosteroids; *: involvement outside radiation field

Reference	Demographics	Comorbidities	Location of RHBLS	Clinical	Time after radiation	Histology	Treatment	Outcome
Trattner (1994)^6^	57F	Not mentioned	Right breast	Clusters of asymptomatic vesicles on the sclerotic area; most of the vesicles had clear content, but some were hemorrhagic	3 years after radiation therapy	Hyperkeratosis; edema of the upper dermis, and hydropic degeneration of the basal layer; inflammatory infiltrate in mid-dermis; lower dermis shows swollen homogenous collagen bundles and fibrosis	None	Spontaneous resolution within 12 months
Petersen (2018)^3^	57F, White	Not mentioned	Right breast, then bilateral breasts and vulva*	Multiple hemorrhagic bullae, regions of induration and shiny white sclerosis with overlying skin atrophy	5 months after completion (only in right breast)	“Features classic for bullous lichen sclerosus et atrophicus (LS&A) in the superficial dermis.” Direct immunofluorescence (DIF) negative.	OCS tapers, TCS	Marked improvement
Bonfill-Ortí (2019)^5^	71F	Not mentioned	Right breast	Flaccid blister containing serosanguineous fluid and small, round, pearly white macules	22 months after radiation therapy	Epidermal atrophy and marked dermal hyalinization with superficial edema; secondary subepithelial vesiculation	Topical tacrolimus	Blister resolved; macules attenuated
Our case	71F, White	Cholelithiasis, hyperlipidemia, carpal tunnel syndrome	Bilateral breasts, then axillae and inguinal*	Hemorrhagic bulla with surrounded by porcelain-white skin	10 years after right breast irradiation; 2.5 years after left>right breast irradiation	Epidermal atrophy with orthokeratosis; pauci-inflammatory subepidermal bulla; band of edematous, homogenized collagen in the papillary dermis with a dense degree of lymphocytic infiltration	OCS taper, TCS	Partial improvement

RHBLS typically presents as painful, hemorrhagic bullae with surrounding sclerotic tissue. Involvement of skin outside the field of radiation, as noted in our patient, can be a feature seen in both radiation-induced LS and morphea but is atypical for other radiation-induced dermatoses [5,6]. If found to have RHBLS, a full body skin exam would be recommended to evaluate for other sites to rule out genital involvement [5]. We favored radiation as the most likely etiology as the lesions started on the breast prior to expanding to areas outside the radiation field as reported in the literature [5,6]; however, friction from wearing a bra could also be a contributory factor [5] but would not explain the involvement outside the treatment area. Clinically, RHBLS can overlap with bullous morphea including hemorrhagic bullae, necessitating a biopsy; however, porcelain-white skin is characteristic of LS. Histologically, morphea is characterized by excessive collagen deposition, which was not seen in our patient [4]. On histology, RHBLS is characterized by epidermal atrophy with orthokeratosis, a band of edematous, homogenized collagen in the papillary dermis with a dense degree of lymphocytic infiltrates, and subepidermal bullae [1,2].

Regarding pathophysiology, koebernization from trauma, including radiation, has been reported [1,3,5]. Only Bonfill-Ortí et al. and our case report any treatment for breast cancer other than surgical intervention and radiation, suggesting that hormonal therapy is less likely to be the underlying trigger for RHBLS [2]. Radiation is thought to induce a TH2 immune response leading to increased fibroblast activity and sclerosis by upregulating IL-4 and TGF-beta, which can cause LS [5]. It is hypothesized the bullae that develop are because of alteration of the lymphatic vessels secondary to radiation [1]. The radiation regimen, including dose, fractionation, and boost, does not appear to correlate with the risk of developing RHBLS [5]. Treatment for RHBLS includes oral or high-potency topical corticosteroids as initial therapy [1,5]. Other therapies include topical calcineurin inhibitors, intralesional steroid injections, methotrexate, and phototherapy [1,2]. Symptoms and bullae have shown improvement in as little as three weeks [1].

Other diagnoses to consider include chronic radiation dermatitis and angiosarcoma. Chronic radiation dermatitis can also present with atrophic areas after radiation, but histopathology shows atypical fibroblasts scattered singly in a background of thickened collagen bundles with superficially located dilated vessels containing swollen endothelial cells [7-9]. Angiosarcomas can occur postradiation and may present as violaceous plaques or nodules, but histopathology highlights an underlying irregular vascular process [9,10].

## Conclusions

To our knowledge, this is the fourth definitive case of RHBLS reported. This report aims to raise awareness about RHBLS, a rare but distinct cutaneous sequela of radiotherapy. Given that radiotherapy is widely used as a primary and adjuvant treatment for cancer, early consideration of RHBLS is advised in patients with a history of radiotherapy who present with sclerotic skin and hemorrhagic bullae, even years after radiotherapy treatment. This case emphasizes the need for increased clinical vigilance and early intervention to prevent further complications. Given the small sample size of only four, including this one, reported cases in the literature, and limited conclusions, it is difficult to generalize the findings, so caution is needed when interpreting results. Continued research and collection of additional cases of RHBLS are necessary to strengthen the evidence and allow the development of clearer guidelines for the diagnosis and management of this condition. Larger case studies or systematic reviews would also help to better understand this condition.
